# Management of a severe skeletal Class II hyperdivergent patient with asymmetric missing teeth with lingual appliances and mandibular osteotomy: A case report

**DOI:** 10.1097/MD.0000000000040843

**Published:** 2024-12-06

**Authors:** Thi Ngoc Anh Tran, Thuy Anh Nguyen, Giao Hoang Nguyen, Viet Anh Nguyen

**Affiliations:** a Faculty of Dentistry, VNU Hanoi-University of Medicine and Pharmacy, Hanoi, Vietnam; b Private Practice, Viet Anh Orthodontic Clinic, Hanoi, Vietnam; c Faculty of Dentistry, PHENIKAA University, Hanoi, Vietnam.

**Keywords:** angle Class II malocclusion, case report, fixed orthodontic appliances, mandibular osteotomy, orthodontic anchorage procedures

## Abstract

**Rationale:**

Orthognathic surgery cases are inherently challenging to treat with lingual appliances due to the complexities of orthodontic management and difficulties in achieving intermaxillary fixation during surgery. This challenge is further amplified in cases involving asymmetric space closure, such as those with a missing molar on one side and a premolar on the other, a scenario not yet documented in the literature. This case report presents the orthodontic–orthognathic management of an adult patient requiring space closure of asymmetric missing lower teeth.

**Patient concerns:**

A 30-year-old female patient presented with a severe skeletal Class II patient with a retruded mandible, hyperdivergent facial pattern, excessive overjet and overbite, and asymmetric missing lower teeth.

**Diagnosis:**

The patient was diagnosed with a Class II malocclusion and asymmetric missing mandibular posterior teeth on a skeletal hyperdivergent Class II relationship.

**Interventions and outcomes:**

The treatment involved orthodontic decompensation with fixed lingual appliances in combination with mandibular advancement osteotomy and genioplasty. Mini-screws were utilized for anchorage control during asymmetric space closure in the presurgical stage and entire lower arch distalization in the postsurgical stage. A significant facial esthetic and functional improvement was achieved posttreatment.

**Lessons:**

The combination of mini-screws and lingual appliances may offer effective anchorage management in both presurgical and postsurgical orthodontic stages for optimal orthodontic–orthognathic treatment outcomes. This approach allows for precise tooth movement and control during space closure in the presence of challenging asymmetric missing tooth patterns.

## 1. Introduction

An estimated one-third of all orthodontic patients are treated for Class II malocclusion to improve both function and esthetics.^[1]^ This condition may be caused by different types of relationships between bones and teeth including maxillary excess, mandibular deficiency, or both the maxilla, mandible, and teeth. Treatment options for Class II malocclusions vary depending on the growth stages, anteroposterior discrepancies, facial appearance, airway, and patient compliance.^[2]^ When there is an excessive discrepancy between the maxilla and the mandible, camouflage treatment can lead to periodontal deteriorations such as gingival recession in the lower anterior incisor, root resorptions, facial esthetic worsening, and occlusal instability.^[2–4]^ Therefore, orthodontic treatment combined with orthognathic surgery is often required in adult patients with severe skeletal Class II to improve both the occlusion and facial profile.^[5,6]^ Presurgical orthodontic treatment is aimed at decompensating teeth by moving teeth to a proper position in relation to skeletal bases.

Orthognathic surgery cases are categorized as difficult to treat with lingual appliances due to the complexity of the orthodontic management and difficulties in obtaining intermaxillary fixation during surgery.^[7,8]^ The difficulty level increases when there is a combination of anteroposterior, transversal, and vertical discrepancies or asymmetric missing teeth. However, lingual appliances are still indicated in highly esthetic-demanding adult patients with severe skeletal discrepancies who cannot adapt to labial appliances. The treatment results of the lingual orthodontic and orthognathic combination have been demonstrated to be satisfactory in several complex malocclusions.^[5,6,9–11]^ It is noteworthy that the orthodontic preparation in these cases was limited to non-extraction^[5,9–11]^ or symmetric extraction^[6]^ approaches, with no documented instances of asymmetric extraction being utilized. However, asymmetric space closure may be necessary in cases with a missing molar on one side and a premolar on the other. This approach, though sometimes required, can pose challenges during the presurgical orthodontic phase. Specifically, achieving three-dimensional control of tooth movement to attain an ideal and harmonious occlusion in both the transverse and sagittal planes can be more complex. Furthermore, achieving postoperative midline balance and facial symmetry can be particularly challenging, demanding meticulous planning and adjustments throughout the presurgical orthodontic phase and postsurgical refinement.

This case report presents the orthodontic–orthognathic management of a severe skeletal Class II patient with a retruded mandible, hyperdivergent facial pattern, excessive overjet and overbite, and asymmetric missing lower teeth. The treatment involved orthodontic decompensation with fixed lingual appliances in combination with mandibular advancement osteotomy and genioplasty.

## 2. Case presentation

### 2.1. Diagnosis and etiology

A 30-year-old female patient presented to our clinic with chief complaints of mouth protrusion and missing teeth in the lower arch. She strongly desired an invisible treatment appliance.

**Figure 1.**
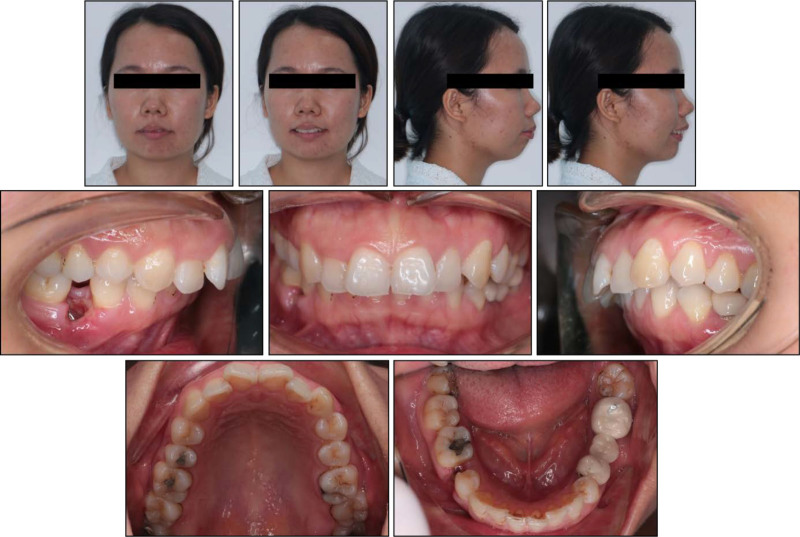
Pretreatment facial and intraoral photographs.

Upon the frontal view, the patient exhibited a long lower facial third with chin deviation to the left. The lateral view showed a convex profile with severe chin retrusion and lip incompetence. No sign of a temporomandibular joint disorder was detected (Fig. 1). The patient also reported snoring during sleep.

**Figure 2.**
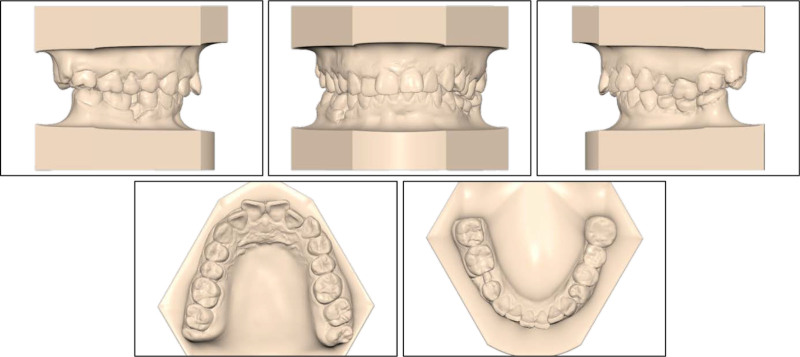
Pretreatment study models.

On the intraoral examination, the patient had a full-cusp Class II canine relationship on both sides, a full-cusp Class II molar relationship on the left side, and a Class I molar relationship on the right side (Fig. 2). The upper and lower arch forms were ovoid with normal transversal arch widths. The patient had an excessive overjet of 6.9 mm and a deep overbite of 5.1 mm (80%). There was moderate crowding with an arch-length discrepancy of 5.8 mm in the upper arch and mild crowding with an arch-length discrepancy of 1.1 mm in the lower arch. Her mandibular right second premolar and left first molar were extracted. The missing mandibular left first molar was restored with a three-unit porcelain-fused-to-metal bridge, showing chipping. The upper midline coincided with the lower midline and with the facial midline.

**Figure 3.**
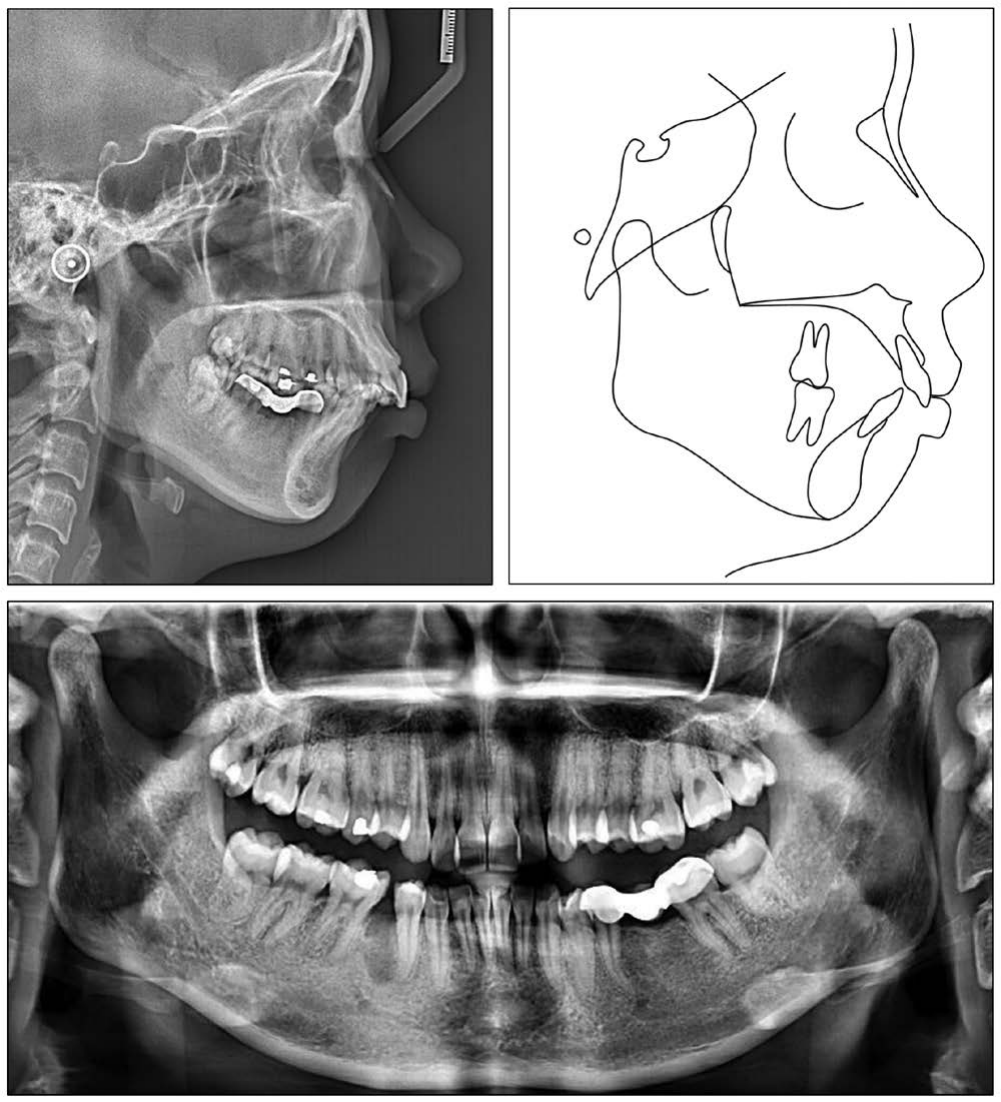
Pretreatment panoramic and cephalometric radiographs and tracing.

The lateral cephalometric analysis revealed a severe skeletal Class II relationship (point A-nasion-point B (ANB): 8.4°) with a retruded mandible (B–N perpendicular, -11.5^o^) and hyperdivergent facial pattern (Frankfort mandibular: 29.2°) (Table 1). The upper incisor’s inclination was in the normal range (upper incisor to sella-nasion: 100.8°) while the lower incisors were proclined (incisor mandibular plane angle (IMPA): 107.3°). Both the upper and lower lips were protruded (upper lip to E-line, 4.4 mm; lower lip to E-line, 5.8 mm). The panoramic radiograph showed the presence of all teeth except the mandibular right second premolar and left first molar (Fig. 3). The patient was diagnosed with a Class II malocclusion and asymmetric missing mandibular posterior teeth on a skeletal hyperdivergent Class II relationship.

**Table 1:** Cephalometric measurements.

	Pretreatment	Presurgery	Posttreatment	Norm
Skeletal				
SNA (°)	84.9	85.0	85.2	81.1 ± 3.7
SNB (°)	76.5	76.7	80.4	79.2 ± 3.8
ANB (°)	8.4	8.3	4.8	2.5 ± 1.8
FMA (°)	29.2	29.3	31.3	25.0 ± 4.0
A to N perpendicular (mm)	1.4	1.3	1.3	0.4 ± 2.3
B to N perpendicular (mm)	-11.5	-11.7	-5.7	-3.5 ± 2.0
Dental				
Upper incisor to SN (°)	100.8	98.4	94.8	105.3 ± 6.6
Upper incisor to NA (°)	15.9	13.4	9.6	22.0 ± 5.0
Upper incisor to NA (mm)	3.7	2.6	1.5	4.0 ± 3.0
IMPA (°)	107.3	100.7	91.0	90.0 ± 3.5
Lower incisor to NB (°)	39.4	32.9	28.8	25.0 ± 5.0
Lower incisor to NB (mm)	9.1	6.7	6.1	4.0 ± 2.0
Interincisal angle (°)	116.3	125.4	136.9	128.0 ± 5.3
Upper incisal display (mm)	3.2	2.0	1.5	2.5 ± 1.5
Soft tissue				
Upper lip to E-line (mm)	4.4	2.8	-1.2	0.0 ± 2.0
Lower lip to E-line (mm)	5.8	2.4	-1.4	0.0 ± 2.0
Upper pharynx (mm)	6.9	7.0	8.6	8.7 ± 2.2
Lower pharynx (mm)	7.6	7.8	11.6	8.4 ± 2.5

ANB = point A-nasion-point B, FMA = Frankfort mandibular angle, IMPA = lower incisor mandibular plane angle, NA = nasion-point A, NB = nasion-point B, SN = sella-nasion, SNA = sella-nasion-point A, SNB = sella-nasion-point B, SN-MP = sella-nasion to mandibular plane.

The following treatment objectives were established: (1) to reduce the lip protrusion and improve the skeletal Class II relationship and chin deficiency, (2) to achieve Class I canine relationships on both sides, (3) to eliminate the severe overjet and deep overbite and (4) to close spaces of missing teeth in the lower arch.

### 2.2. Treatment plan

The first treatment option was a non-extraction strategy including space closure in the lower arch and extraction of first premolars with mini-screw anchorage in the upper arch for maximal retraction of the upper incisors. However, this option would result in retroclination of the upper incisors due to their normal initial inclination.

The second treatment option was an orthodontic–orthognathic combined treatment including extraction of upper second premolars. The presurgical orthodontic phase aims to align the upper arch, level the lower curve of Spee, and retract the proclined lower incisors to create an adequate overjet for mandible advancement.

Considering the severity of the skeletal discrepancy and the initial upper incisor inclination, the patient chose the second option with fixed lingual appliances.

### 2.3. Treatment progress

**Figure 4.**
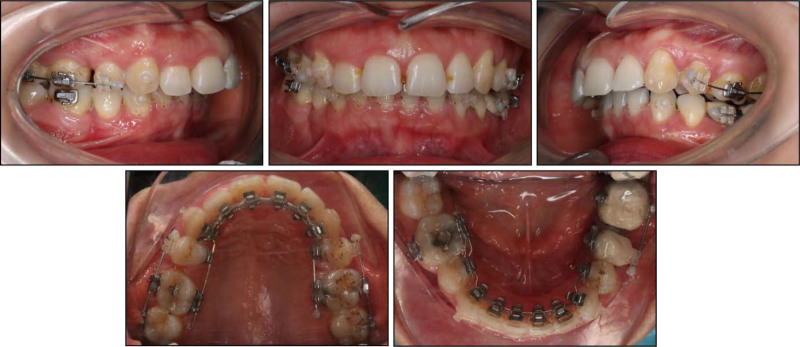
Space closure during presurgical orthodontic treatment.

The orthodontic treatment started by bonding all teeth with 0.018” × 0.025” pre-adjusted lingual appliances (DLB, Dentos, Korea) using double vacuum-formed indirect bonding trays.^[12]^ The leveling and alignment stage was performed by using a sequence of round and rectangular nickel-titanium archwires including 0.012”, 0.014”, 0.016”, and 0.016” × 0.022” wires. After 5 months of treatment, the maxillary second premolars were extracted under local anesthesia. Space closure was achieved using sliding mechanics with 0.016” × 0.022” stainless steel archwires in both arches. A 15-degree pretorqued archwire in the upper arch enabled bodily retraction of the incisors to prevent lingual tipping, as the upper incisors were already uprighted. Meanwhile, in the lower arch, a standard archwire facilitated lingual tipping of the lower incisors (Fig. 4).

**Figure 5.**
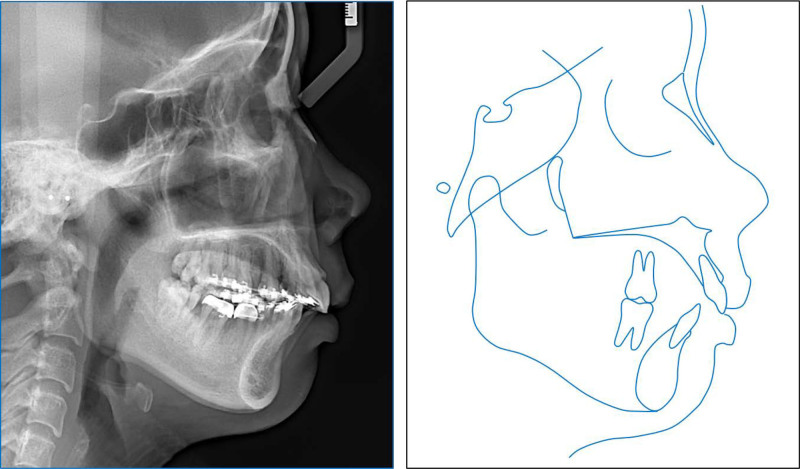
Presurgical cephalometric radiograph and tracing.

During space closure, some labial brackets were bonded to correct crown tipping into extraction spaces according to the crossover technique.^[13]^ Additionally, a mini-screw (diameter 2.0 mm; length 12 mm; Hi-fix, Medico, Korea) was inserted into the left mandibular buccal shelf to correct the right-deviated lower midline with a retraction force of 150 g. The presurgical orthodontic phase was completed in 20 months resulting in a leveled lower curve of Spee, retracted lower incisors (IMPA, 100.7°), well-aligned dental arches, and an adequate overjet of 8.1 mm for surgical correction (Fig. 5).

**Figure 6.**
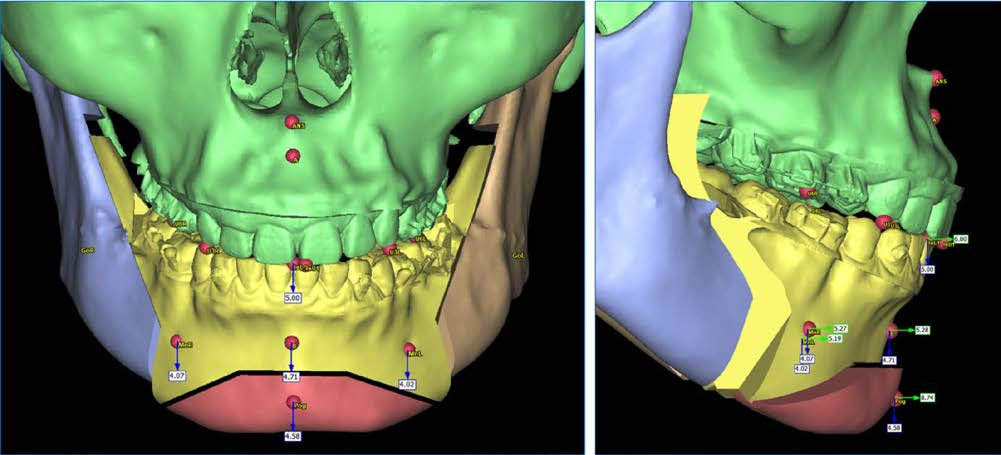
Virtual orthognathic surgery plan.

Before surgery, a digital impression and a cone beam computed tomography were taken and imported into a virtual planning software (Proplan CMF, Materialise, Leuven, Belgium). A bilateral sagittal split osteotomy combined with genioplasty was planned, in which the mandible was advanced by 5.3 mm and the chin was advanced by 3.5 mm (Fig. 6). Surgical guides were exported and printed with a digital light processing 3-dimensional printer (Pro 55S, SprintRay, CA) and a surgical guide resin (Surgical Guide 3, SprintRay, CA). The orthognathic surgery was performed under general anesthesia. During the surgery, the left mandibular buccal shelf mini-screw was removed as it interfered with the placement of the bone fixation plate. The intra-operation intermaxillary fixation was achieved with 4 mini-screws placed between lateral incisors and canines. The removal of intermaxillary fixation took place immediately after the surgery.

**Figure 7.**
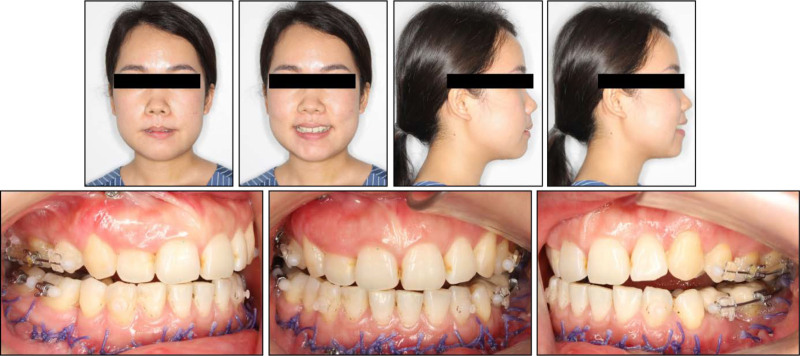
Postsurgical facial and intraoral photographs.

The postsurgical orthodontic phase commenced 2 weeks after the orthognathic surgery (Fig. 7). A slight Class III malocclusion and edge-to-edge bite developed postsurgically. Therefore, 2 mini-screws were inserted into the mandibular buccal shelf bilaterally for entire lower arch distalization with a force of 150 g per side. Interproximal reduction was performed on the upper and lower incisors, removing approximately 0.2 mm of enamel per tooth side, to reduce gingival black triangles as requested by the patient. The postsurgical orthodontic phase took 6 months, and the total active treatment time was 26 months. After the appliance removal, the old porcelain-fused-to-metal crowns were replaced with all-ceramic crowns. Fixed retainers were placed in both arches in combination with Essix retainers for nighttime wear.

### 2.4. Treatment results

**Figure 8.**
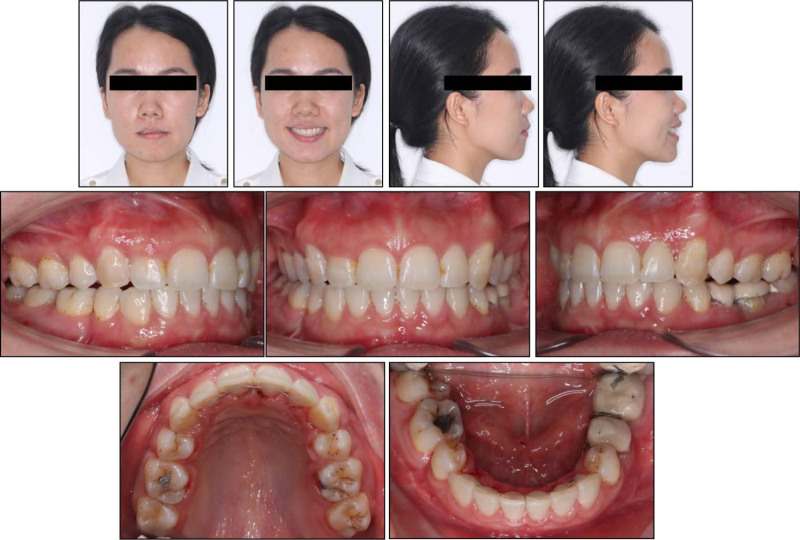
Posttreatment facial and intraoral photographs.

**Figure 9.**
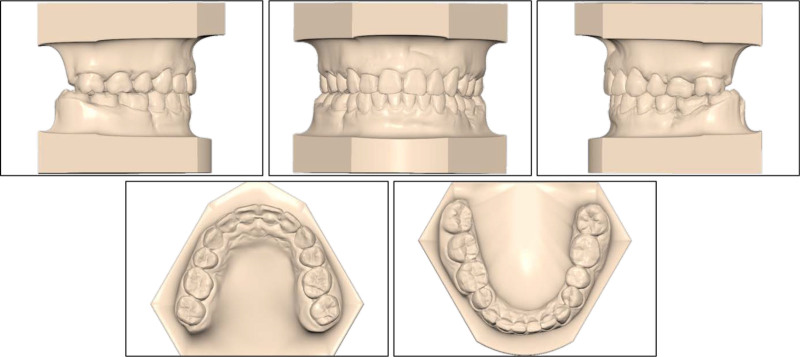
Posttreatment study models.

Posttreatment records show that all treatment objectives were achieved with a well-aligned dentition and improved facial esthetics (Fig. 8). A super Class I canine was obtained on both sides with a normal overjet and overbite. The lower curve of Spee was leveled and all extraction spaces were completely closed (Fig. 9). The retruded mandible, left-deviated chin, and convex profile were significantly improved. Additionally, the patient reported an improvement in the snoring condition.

**Figure 10.**
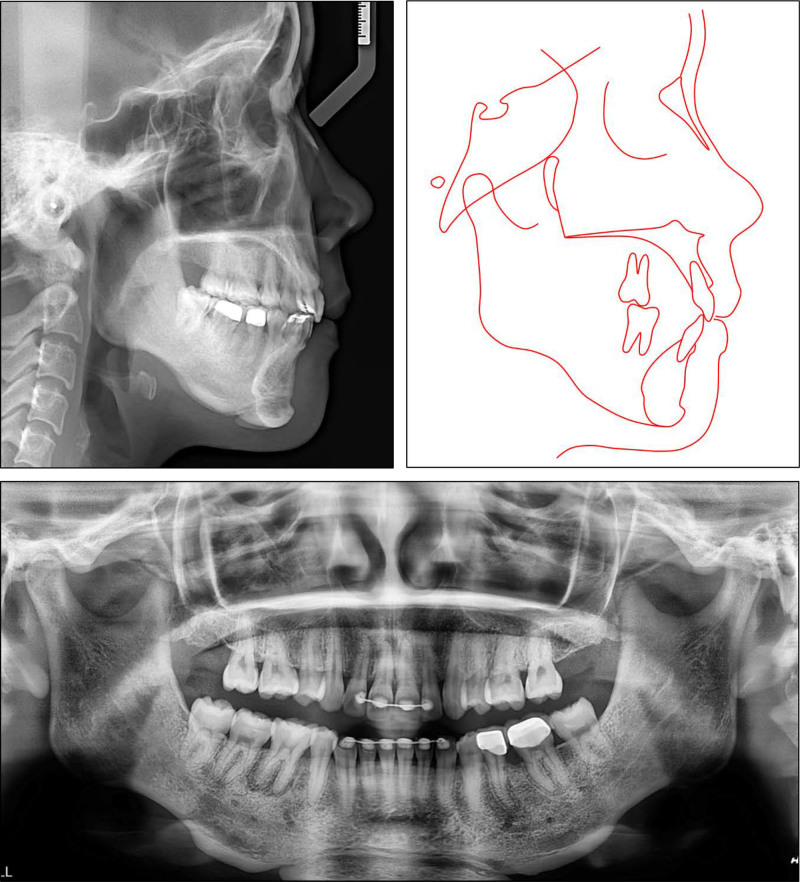
Posttreatment radiographs and tracing.

The lateral cephalometric analysis confirmed the improvement of the skeletal Class II relationship (ANB: 4.8°) and the mandibular retrusion (B–N perpendicular: -5.7 mm). The lower incisor’s inclination was normalized (IMPA: 91.0°). The lip protrusion was corrected (upper lip to E-line: -1.2 mm; lower lip to E-line: -1.4 mm). The pharyngeal airway space was also improved (upper pharynx: 8.6 mm; lower pharynx: 11.6 mm). The panoramic radiograph showed adequate root parallelism and no sign of root resorption (Fig. 10).

**Figure 11.**
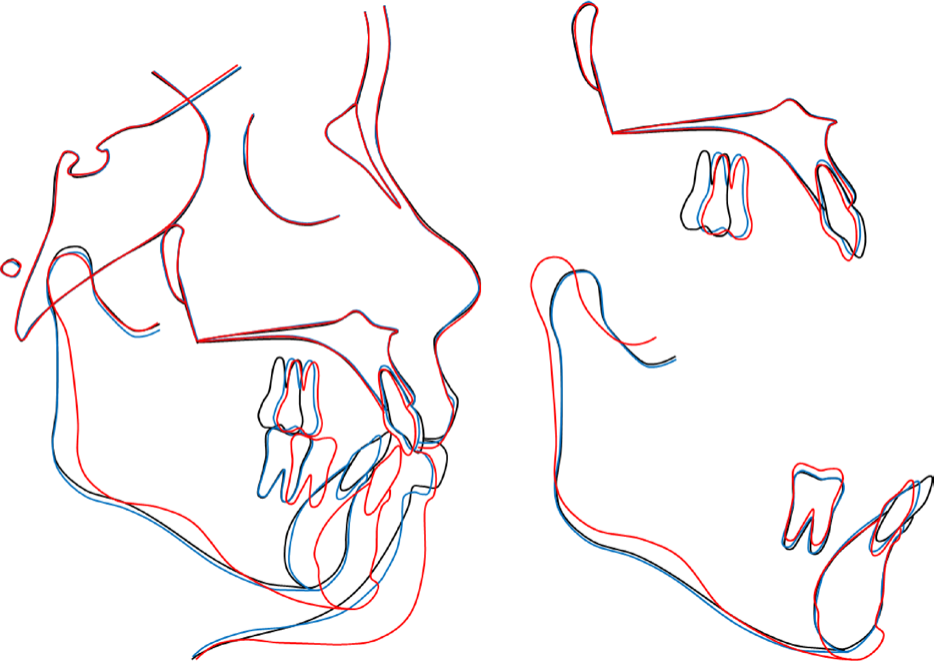
Overall and regional cephalometric superimpositions: black, pretreatment; blue, presurgery; red, posttreatment.

**Figure 12.**
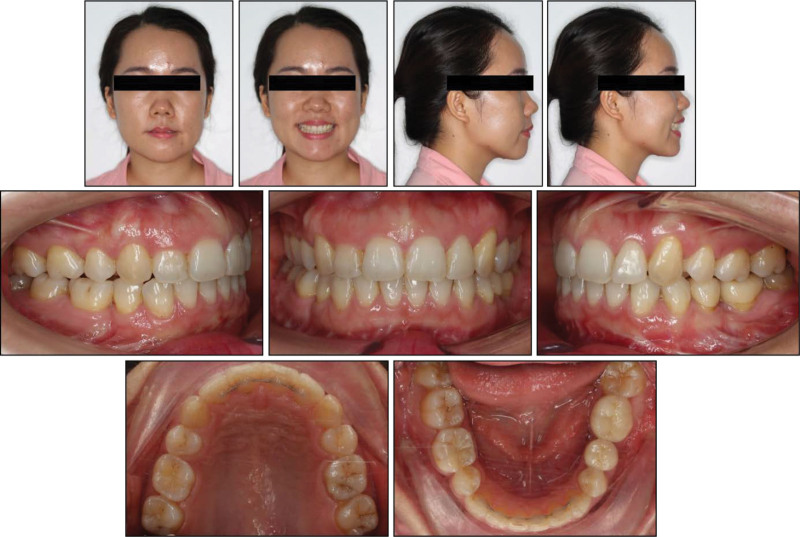
One-year post-retention facial and intraoral photographs.

The general and regional cephalometric superimpositions showed the mesialization of the upper molars, the slight lingual tipping of the upper incisors, the significant lingual tipping and intrusion of the lower incisors, and the advancement of the mandible and chin (Fig. 11). The one-year post-retention records showed the stability of the treatment result and a further improvement of the occlusal settling (Fig. 12). The patient was satisfied with the treatment outcomes and the esthetics of the lingual appliances during treatment.

## 3. Discussion

In the present case report, a severe skeletal Class II malocclusion with retruded mandible and hyperdivergent facial pattern was corrected by a combination of orthodontic and orthognathic management with lingual appliances, mini-screws, and mandibular advancement osteotomy. Space closure was performed with minimal anchorage in the upper arch and maximal anchorage in the lower arch to decompensate the lower incisors and create a proper overjet for skeletal movements. A mini-screw was placed on the left mandible to move the lower dental midline to the left, facilitating the repositioning of the chin to the right during the orthognathic surgery. Additionally, mini-screws were used in the postsurgical orthodontic phases to correct the Class III malocclusion, possibly resulting from an over-advancement of the mandible during surgery.

With recent advancements in digital dentistry and temporary anchorage devices, orthognathic cases can be treated routinely in combination with lingual orthodontics. Lingual appliances have been demonstrated to achieve an accuracy of predetermined tooth movement of more than 75%.^[14,15]^ The difficulty in performing intermaxillary fixation during surgery with lingual appliances can be overcome with temporary anchorage devices.^[8]^ Digital orthognathic planning and the fabrication of surgical guides can visualize the surgical outcomes and move the bone to the intended position. The present case report utilized double vacuum-formed indirect bonding trays, which have been proven to have high lingual bracket transfer accuracy.^[16]^

The mandibular advancement improved not only the facial esthetics but also the occlusal function and upper airway.^[17]^ Both the upper and lower pharyngeal airway spaces were enlarged, resulting in an improvement in the patient’s snoring condition. Additionally, the chin augmentation may contribute to lip competence, facilitating nose breathing.^[18,19]^ These improvements would not have been achievable if a camouflage treatment option had been pursued.

In this patient, the chipped porcelain-fused-to-metal bridge was intended to be replaced after the orthodontic treatment to achieve proper adaptation to the new occlusion. The brackets were bonded to the old porcelain after roughening with a diamond bur, etching with hydrofluoric acid in 120 seconds, and priming with silane.^[20]^ The bonding strength between the brackets and porcelain was adequate without bracket failure during the entire treatment.

This study has inherent limitations due to its case report design, potentially limiting the generalizability of findings regarding the combination of mini-screws and lingual appliances in the orthodontic management of orthognathic patients. Another limitation is the reliance on an analog indirect bracket bonding procedure and mushroom archwires, rather than employing a more contemporary digital workflow and straight archwires.

## 4. Conclusions

A significant facial esthetic and functional improvement was achieved by lingual orthodontic treatment and orthognathic surgery in a severe skeletal Class II hyperdivergent patient with asymmetric missing teeth. The combination of mini-screws and lingual appliances may offer effective anchorage management in both presurgical and postsurgical orthodontic stages for optimal orthodontic–orthognathic treatment outcomes.

## Author contributions

**Conceptualization:** Thi Ngoc Anh Tran, Viet Anh Nguyen.

**Data curation:** Thuy Anh Nguyen, Giao Hoang Nguyen, Viet Anh Nguyen.

**Methodology:** Viet Anh Nguyen.

**Writing – original draft:** Thi Ngoc Anh Tran, Thuy Anh Nguyen, Viet Anh Nguyen.

**Writing – review & editing:** Thi Ngoc Anh Tran, Viet Anh Nguyen.
